# Individual and community level factors for modern contraceptives utilization among reproductive aged women in Amhara region, Mixed effect multi-level modeling, Data from Mini-EDHS, 2019

**DOI:** 10.1186/s40834-023-00256-6

**Published:** 2023-11-28

**Authors:** Fassikaw Kebede Bizuneh, Tsehay Kebede Bizuneh, Seteamlak Adane Masresha, Berihun Mulu Yayeh

**Affiliations:** 1 School of public health, College of health science, Woldia University, North East, Ethiopia; 2 Department of Geography, Faculty of social science, Bahir Dare University, Bahir Dar, Ethiopia

**Keywords:** Modern contraceptive, Sexually active women, Individual factors, Amhara, Ethiopia

## Abstract

**Background:**

Modern contraceptive has been identified as a key strategy to control unintended pregnancy, protect the health of the mother and child, and promote women wellbeing. Despite this and increasingly wider availability of modern contraceptives, however, there are still high levels of unmet need for birth interspacing and contraceptive use in Amhara regions. This study aimed to identify factors associated with the enhancing of modern contraceptives utilization among reproductive aged women in Amhara region, using a mixed effects multilevel modeling data from mini-EDHS 2019.

**Methods:**

A community-based cross-sectional study was conducted among 711(wt = 100%) samples of reproductive-aged women. The data were interviewed by trained data collectors using a semi-structured questionnaire for the final mini-EDHS 2019 data set. A multilevel binary logistic regression model was fitted to identify the enhancing factors for modern contraceptive utilization Adjusted Odds Ratio (AOR) with 95% Confidence Intervals (CI) was used to identify factors associated with utilization.

**Result:**

The median age of the participants was 31 with (IQR ± 13) years. The overall modern contraceptive utilization was 42.3% (95%CI: 38.7; 46.1). Individual and community factors accounted for 21.4% of the variation in modern contraceptive utilization at the cluster level. Being age 25 years (AOR = 12.99; 95%CI: 4.5–37.2), 26–35 years (AOR = 8.8, 95%CI: 3.25- 24), 36–45 years (AOR = 5.6, 95%CI: 2.2–16.2), being married (AOR = 4.2, 95%CI: 2.21–6.97), educated women (AOR = 11.6, 95%CI: 3.22–40.4), and being middle-economic class (AOR = 3.03; 95%CI: 1.87–4.91) were identified as individual enhancing factors. Whereas, being urban resident (AOR = 5.19; 95%CI: 5.19: 41.7) and having media exposure (AOR = 1.5; 95%CI: 1.58–3.7) were community-level enhancing factors for modern contraceptive utilization.

**Conclusion:**

Compared to earlier studies, in Amhara region, a lower prevalence rate of modern contraceptive utilization was reported. The variation in utilization at the cluster level, 21.4%, was attributed to individual and community-level factors. Healthcare providers should prioritize raising awareness about contraceptive side effects to encourage new users and decrease the number of individuals who discontinue contraceptive methods.

## Introduction

Family planning refers to the ability of individuals or couples to make informed decisions and actively control the number of children they have, as well as the timing and spacing of their births, in order to align with their desired preferences [1, 2]. Many reproductive-age women do not have access to information and services; they need a cheap and effective strategy to promote their sexual and reproductive health. This appropriate utilization of modern contraceptives enables the realization of this goal [3]. Modern contraceptive utilization helps to reduce poverty, increase gender equity, prevent the spread of sexually transmitted infections, and reduce maternal, infant, and childhood mortality [3, 4]. The provision of adequate information and providing different modern contraceptive types can avert 32% of all maternal deaths and loss of 10% of childhood death [5]. The utilization of modern contraceptives, such as intrauterine contraceptive devices (IUCDs), implants (such as Implanon, Jadelle, and Sinoplant), injectable combined oral contraceptives, emergency pills, and female condoms, played a significant role in preventing approximately 308 million unintended pregnancies and averting 3 million deaths caused by unsafe abortions [6, 7].

Globally, in 2019, 172 million women are not using any method of contraception even though they desire to avoid pregnancy [8]. The overall modern contraceptive utilization prevalence rate (CPR) was 56%in the globe. This has significant variation in both developed and developing nations, 62%and 54%, respectively. Specifically, CPR is low as 4.6% in South Sudan and as high as 72.1% in Canada [3, 9, 10]. The African continent achieves only 26% of the globe [11–13] and the use of modern contraceptives was hampered by several factors, including religious opposition, spouse opposition, and fears about adverse effects [7, 14, 15]. In underdeveloped countries like Ethiopia, policymakers are increasingly concerned about the rising rates of contraceptive discontinuation following its initiation. Fear of adverse effects and inadequate information are significant factors contributing to abortion after the commencement of contraception, which poses a pressing issue [16]. The reason for discontinuation varies by contraceptive method and is broadly categorized as method failure (fear of side effects), fertility-related (desire for son children), partner’s disapproval, and lack of adequate awareness were some of the factors for discontinuation after starting [17, 18].

The Ethiopian government has implemented FP2030 strategy to enhance the utilization of voluntary family planning, aiming for a 17% increase by 2030. Nevertheless, utilization has impacted by socio-demographic factors of women, which contribute to an 8.6% variation in its usage [7, 11, 19]; but socio-demographic factors of women accounted 8.6%varration in utilization [20]. In Ethiopia, the overall unmet need of family planning was 29.78% [8, 10]. Factors such as the early marriage of a girl have a significant barrier to reproductive health services and cause low confidence in using contraceptives [4, 21]. The problem is more worsening in this region, and the girl is enforced and to have a baby soon after marriage without sufficient economy [7, 11, 19]. According to prior studies, one out of every four married women who desired to avoid pregnancy did not use contraception and had at least one unintended pregnancy [11, 19, 22]. Identifying modern contraceptive use-enhancing factors among sexually active women increased the shifts in fertility rate, accelerated productivity, and quality of life on women’s health [23]. According to a previous compositional study findings in Ethiopia, socio-economic change contributes 45% modern contraceptive uses [23] through positive attitude of household husbands and coupled discussion [23]. Identifying enhancing’s factors for modern contraceptive utilization and reducing fertility rate is a prioritized set for fertility transition to bring economic growth in a given nation and support unified- economic growth theory [24, 25]. Therefore, this study aimed to investigate factors associated with modern contraceptives utilization among reproductive aged women in Amhara region, mixed effects multilevel modeling data from mini-EDHS 2019.

### Research question

1. What is the individual-level factors associated with modern contraceptive utilization among reproductive-aged women found in Amahra Region?
2. What are community-level enhancing factors to increase modern contraceptive utilization for reproductive-aged women in the Amhara region?

## Methods

### Study setting

The data for this study were accessed from the EDHS official database; which is conducted at the national level in Ethiopia from March 2019 to June 2019 and a detailed explanation is publicly available at https://dhsprogram.com/data/ with a [26]. The survey is usually conducted at five–year intervals in the country and four consecutive DHS surveys were conducted in Ethiopia in 2000, 2005, 2011,2016, and mini EDHS was conducted on 2019 [27].

### Study design

A community-based cross-sectional survey was employed among 711 sexually active reproductive-age women in the Amhara region.

### Sampling size

In the Ethiopian Mini-demographic and health survey of 2019, there were Eleven (11 data collection regions), and the Amhara region was the one that contributed 926 participants women overall 86,794 national level representatives [28]. After editing and cleaning the data set by extracting 962/8679 (11.05%) regional participants, only 711(Wt = 100%) sexually active reproductive-aged women were included after excluding pregnant and mothers having ≤ 4 week-aged babies.

### Sampling procedure

Since EDHS was a national-level enumeration of data and has predefined data collection sites in 11 regions and used equal proportional sample selection principles. Due to this 926 predefined data collection, the household was previously marked for the final survey and interviewed by each housewife of the household or / the head [26, 28].

### Sources population

All sexually active reproductive-age women in the Amhara region were the source population.

### Inclusion criteria

All sexually active reproductive-aged (15 to 49 years) women during this regional survey were included in the final interview.

### Exclusion criteria

All Pregnant women, mothers with dyads ≤ 1 month of age, and traditional contraceptive utilizer were excluded from the final analysis.

### Variables

#### Dependent variables

The outcome variable is modern contraceptive use, which is defined as (Yes, used /Not used). The listed types of contraceptives(intrauterine contraceptive devices, injectable pills, implants, female condoms, lactation, amenorrhea, and emergency contraception) were considered for the final data collections of this survey [23].

#### Independent (explanatory) variables

During the final data analysis, two components; i.e., individual and community-level factors were evaluated on sexually active reproductive-aged women in Amhara region.

##### Individual-level characteristics (level I)

which includes maternal age (15–49), education (no education, primary, secondary, and higher), religion (protestant, Muslim, Catholic, Orthodox, and others), Number of live children in five years, Wealth index (Poor, middle, rich, and too rich), media exposure (yes, no), age of children, and family size of the participant were assessed.

##### Community-level factors (level II)

Community-level factors of women include residents (urban and rural), media exposure to family planning (Radio, TV, Newspaper, Mobile messages), and categorized as (Yes/No).

### Operational definition

#### Mass media exposure

Mini-EDHS 2019 asked reproductive-aged women whether they have heard or exposed messages on modern contraceptive utilization from television, radio, newspaper, and mobile messages about family planning during the last months before the interview day. Given that these four variables were highly correlated, and we decided to create a variable called ‘exposure to mass media messages’ with this, we coded as “yes” if respondents were exposed to messages from at least one of the four media outlets (0 = No &1 = Yes) and considered as community levels factors [29].

### Data quality control

Before the dataset was accessed, the first proposal was submitted to the Demographic and Health Surveys (DHS) program to use the mini-EDHS 2019 dataset, the Mr.FK and data set were accessed and cleaning, and editing were followed using STATA version 17. During data collection, women aged 15–49 years in selected households were interviewed by a trained data collector by Ethiopian Demographic and Health Survey (EDHS)research teams members.

Trained data collectors who are employed by EDHS offices employed computer-assisted personal interviewing (CAPI) data collection systems [29].

### Statistical analysis

The data were extracted, cleaned, recorded, and analyzed using STATA version 17 (SE). The EDHS samples were not self-weighted due to the non-proportional allocation of samples from different regions as well as urban and rural areas, and possible differences in response rates. Thus, the data were weighted before doing any statistical analysis to restore the representativeness of the sample and get a reliable estimate and standard error [29].

### Modeling building

Since our objective is to determine the multilevel enhancing factors for modern contraceptive utilization of reproductive-aged women through multilevel binary logistic regression, we used an advanced model to overcome the violation of the independence of observation of EDHS data. We first estimated the null model (Model I) containing only the outcome variables, which is the utilization of modern contraceptives (Yes/No).

The second (Model II) is a multivariable adjustment for individual-level variables, which included a p-value of less than 0.25 in the bivariable regression model. Which contain all individual-level factors, variables that were significant candidates transferred to multivariable with a criterion of P < 0.25 in the second model were considered as candidates for the final model (Model IV) regression. Moreover, in the third stage, community-level variables with dependent variables were regressed and selected when variables ≤ 0.25 were considered candidates for the final model (Model IV) regression.

In the fourth model (Model IV), both individual and community-level variables were assessed simultaneously with the dependent variables, which were captured from each model with statistically significant candidate transferred criteria for final model fitness. During this time, the adjusted odds ratio (AOR) and confidence interval (95%CI) was used to measure the association of the covariate with outcome variables after model comparison and adjustment for confounding.

Proportional Changes in the Community-level variance (PCV), and Median Odds Ratio (MOR) were calculated at this stage. PCV measures the proportional change in the community community-level variance (Model I) and the subsequent model (Model II). The MOR aimed to translate the community-level variance into the widely used odd ratio scale, which has a consistent and intuitive interpretation [8]. MOR is defined as the median value of the odds ratio between the area with the highest contraceptive utilization and the area with the lowest contraceptive utilization when randomly picking out the two clusters [30]. The Final multilevel binary logistic regression model was expressed in equation form as follows;

Logit (p) = log (p/1 − p) = β0j + β1jX1ij + β2jX2ij + ……βqjXqij

= since, В0j = γ00 + γ0sZsj + u0j

=Вqj = γq0Xqij + γqsZsjXqij + uqjXqij or

**= Logit(p) = γ00 + γ0sZsj + γq0Xqij + γqsZsjXqij + u0j + uqjXqi.**

Where we have “q” as the explanatory variable at the lower level, and “s” as explanatory variables at the highest level; each in detail expressed as.

j = subscript indicates that this case belongs to the jth group.

ij = subscript indicates that the ith individual within the j^**th**^ group.

γ00 = the overall intercept (fixed part).

Boj = is a random intercept at the community level.

Β&S = were “γ” and “s” regression coefficients.

U0j = Error term of the intercept or deviation from the average intercept.

Uqj = Error term of slope.

βqj or deviation from average slope βq due to level-2 explanatory variable Zsj.

### Model comparisons

The final model tested its fitness based on three model classes; the intraclass correlation coefficient (ICC), the likely hood ratio (LR) test, the Akakian information criteria (AIC), and Bayesian Information Criteria (BIC) test since the model is by nature being nested. The final model III (individual + community) was the best-fit model for this study.

## Result

### Sampled characteristics

Overall, 711(Wt = 100%) sexually active reproductive aged women were included for final analysis. The median age of participant and women was found to be 31(IQR ± 13) years. Nearly one-fourth 275 (38.68%) of the participants were between 26 and 35 years. More than half 459 (64.56%) of those had no formal education, but only 23 (3.23%) of them had a certificate and above. Regarding the respondents’ religious 619 (87.06%) participants, women were orthodox religious followers, but only six (0.8%) of them were catholic religion believers. Whereas, nearly one-third of the respondents (203, 28.55%) and one-fourth (172, 24.19%) were found to be from middle-class and low-income backgrounds, respectively Table 1.

**Table 1:** Socio-Demographic characteristics of sexually active reproductive aged women to identify determents for utilizing modern contraceptives in Amhara region, Mini EDHS 2019

Variable	Categories	Number (Weighted)	Frequency
Age of the women	**≤ 25**	**207**	**29.11**
	**26–35**	**275**	**38.7**
	**36–45**	**190**	**26.72**
	**≥ 46**	**39**	**5.49**
Resident	**Urban**	**92**	**12.9**
	**Rural**	**619**	**87.06**
The educational status	**No education**	**459**	**64.56**
	**Primary**	**197**	**27.7**
	**Secondary**	**32**	**4.6**
	**Higher**	**23**	**3.3**
Wealth index	**Poorest**	**95**	**13.4**
	**Poorer**	**172**	**24.2**
	**Middle**	**203**	**28.5**
	**Richer**	**142**	**19.97**
	**Richest**	**99**	**13.9**
Religion	**Orthodox**	**615**	**86.5**
	**Catholic**	**6**	**0.8**
	**Protestant**	**11**	**1.55**
	**Muslim**	**79**	**11.1**
Mass media message exposure	**Yes**	**114**	**16.03**
	**No**	**597**	**83.97**
Household members	**≤ 4**	**339**	**47.68**
	**5–8**	**342**	**48.1**
	**≥9**	**30**	**4.2**
Number of living children	**No**	**275**	**38.68**
	**1–4**	**428**	**60.2**
	**≥ 5**	**8**	**1.14**
Birth in last three years	**No birth**	**431**	**60.6**
	**One**	**266**	**37.5**
	**>=2**	**14**	**1.97**
Knowledge of family planning	**knows no method**	**30**	**4.22**
	**knows modern method**	**681**	**95.7**

### Information about modern contraceptives

Regarding the information on modern contraceptive majority, 681 (95.78%) of participant women heard about the utilization of modern contraceptives from a health institutions, but only 16.8% of them had media exposure on the types and when to the use of modern contraceptives. Moreover, of the total contraceptive users, 283(94.1%) were married women, but the remaining 13(4.3%) and 3(0.99%) were divorced and widowed contraceptive users’ women, respectively.

### Level of modern contraceptive utilization

At the end of the study, the overall prevalence of modern contraceptives was 42.3% (301/711) (95%CI: 38.7; 46.1) in Amhara region. Majority 174(57.8%) of contraceptive utilizers were used injectable types, and the remaining 65(21.5%) were implants, 8(2.7%) pills and 8(2.7%) IUCD, respectively. On the other hand, 21(6.9%) sexually active reproductive women used periodic abstinence, lactation, amenorrhea, diaphragm, and female sterilization in cumulative.

### Random effects and modern comparison

**As shown in** Table 2, the intraclass correlation of the null model (Model = 1) was found to be 12.3%; which is exclusively 12.3%variation of modern contraceptive uses among reproductive-age women was attributed to the difference in zonal level family planning service variation. Whereas there was 21.4%variation in modern contraceptive utilization. This is evidenced by the significant variability of the intraclass correlation of multilevel regression (ICC = 16.3%), Table 2.

**Table 2:** Random effect analysis and model fitness for assessing factors associated with modern contraceptive utilization among married women in Amhara region, EMDHS, 2019

Radom effect	Model I	Model II	Model III	Model IV
Community variance	**0.214(0.03)**	0.14(*0.054)	0.14(*0.06)	0.13(*0.11)
ICC	**16.3 (9.2–25)**	**20.8(11.3–33.1)**	**22.3(12.7–33.8)**	**27.1(15.9–38.9)**
PCV	**Reference**	**43.9%**	**42.1%**	**53.7%**
MORE	**3.03**	**3.93**	**4.16**	**5.07**

### Factors associated with modern contraceptives

The difference in modern contraceptive utilization was assessed both with individual and community level regression of mixed effects multilevel analysis.

### Individual level factors in model II

We first estimated the null model (Model I), which is containing only the outcome variables (Yes/No) and followed Model II (outcome variables + individual level factors) were regressed. After ascertainment of certain cofounding in the final regression of model II, being aged ≤ 25 years, 11.9 (AOR = 11.9, 95%CI: 3.9–36.9], 26–35 years 8.5 (AOR = 8.5, 95%CI: 2.8–25.7], 36–45 years 3.4 (AOR = 3.4, 95%CI: 1.14–10. 6]being married women were 4.2 (AOR = 4.2, 95%CI: 2.3–7.2) were identified as factors enhanced modern contraceptive utilization as compared with counter groups. Likewise, sexually active reproductive age women being completed primary school, Secondary, and preparatory school were (AOR = 1.8, 95%CI: 1.28–2.6], (AOR = 3.7, 95%CI: 1.65–8.6], and (AOR = 8.02,95%CI: 2.5- 25.31] significantly associated with contraceptive utilization respectively. Moreover, sexually active reproductive-aged women having children aged ≤ 12 months and 13–36 Months after birth had (AOR = 4.9, 95%CI: 1.9–12.8], and (AOR = 6.6, 95%CI: 2.7–15.7) times significantly associated with utilization of contraceptive, respectively.

### Community-level factors in model III

During the final report of Model III, community-level factors were added to assess enhancing factors for modern contraceptive utilization. Accordingly, resident and mass media exposure were significantly associated with contraceptive utilization (AOR = 1.9, 95%CI:1.2–4.1]and (AOR = 14.4[5.19; 41.7] time increased enhancement of modern contraceptive utilization Table 2.

### The final model (model IV)

In the final multivariable analysis, the following individual and community-level factors were associated with modern contraceptive utilization for sexually active reproductive-aged women. After controlling certain confounding in the final model (Model IV), there were two community-level and eight individual-level factors that were enhancing the utilization of modern contraceptives.

Accordingly, age ≤ 25 (AOR = 12.99; 95%CI: 4.5–37.2), 26–35 years (AOR = 8.8, 95%CI: 3.25–24.01), and 36–45 years (AOR = 5.6, 95%CI: 2.2–16.2) were significantly associated with utilization of modern contraceptive as compared with women aged ≥ 46 years.

Moreover, the odds of modern contraceptive utilization were 4.2 (AOR = 4.2, 95%CI: 2.21–6.97) time enhanced by married women as compared with divorced. In this report, the odds of utilizing modern contraceptives were 11.6 (AOR = 11.6, 95%CI: 3.22–40.45) and time improved for reproductive-aged women who had completed preparatory school ‘as compared with women who had no formal education.

The final multivariable analysis the odds of modern contraceptive utilization for women with a middle economic index were 3.03(AOR = 3.03; 95%CI: 1.87–4.91) and time heightened as compared with poor household economic class.

Sexually active reproductive-aged women and having dyads/children with 13 **to** 36 months aged was 3.17 (AOR = 3.17, 95%CI: 1.5–6.63) time enhanced utilizing modern contraceptive methods as compared with mothers with her baby ≥ 37 months of age.

In this report, women who have ≤ 2 families in their house were 2.2(AOR = 95%CI: 1.5–3.23) times the enhanced odds of utilizing modern contraceptives as compared with no children. As a final point, on multivariable regression of the final model, the odds of modern contraceptive utilization were 14.4 (AOR = 5.19; 95%CI: 5.19: 41.7) time enhanced for those sexually active aged women who were from urban residents as compared with from rural resident. Reproductive-aged women with media exposure to family planning were 1.5 (AOR = 1.5; 95%CI: 1.58–3.68) times increased odds of utilizing modern contraceptives than those who had no media exposure seen (Table 3).

**Table 3:** Multivariable multilevel analysis for determinants of modern contraceptive utilization of sexually active reproductive women in Ethiopia, data from Mini-2019 EDHS

Variables	Categories	Model I	Model II AOR (95%CI	Model III AOR (95% CI)	Model IV AOR (95% CI)
Age of women	**≤ 25**		11.9 [3.9 36.9]***		12.99 [4.5- 37.2]***
	**26–35**		8.5 [2.8 25.7 1]***		8.8[3.25- 24.01]***
	**36–45**		3.4[1.14–10. 6]*		5.6 [2.2- 16.2]***
	**≥ 46**		**Ref**		Ref
Marital Status	**Married**		4.2[2.3–7.2]***		4.2[2.21–6.97]***
	**Divorced/Widow**		**Ref**		Ref
Level of Educational	**No education**		**Ref**		Ref
	**Primary**		1.8[1.28–2.6]*		1.36[0.94–1.9]
	**Secondary**		3.7 [1.65 - 8.6]*		2.1[0.95 - 5.30]
	**Higher education**		8.02 [2.5 − 25.31]***		11.6[3.22–40.45]***
Wealth index	**Poor**		**1.18 [0.5 - 1.69]**		1.6 [0.99- 2.73]
	**Middle**		**1.12 [0.9 - 1.8]**		3.03[1.87- 4.91]***
	**Rich**		**Ref**		Ref
Birth in the last 3 years	No		**Ref**		Ref
	One		**1.08[0.03–3.5]**		1.4[0.42- 4.96]
	>=2		**1.8[0.56–6.23]**		1.6 [0.53–5.2]
Household members	**≤ 4**		**1.78 [0.71–4.3]**		1.2[0.77–1.98]
	**5–8**		**1.5 [0.62- 3.7]**		1.17[0.74–1.66]
	**≥ 9**		**Ref**		Ref
Age of Children (N = 586)	**≤ 12 Month**		6.2 [3.2 - 12.8]*		1.6 [0.69–3.8]
	**13–36 Month**		7.6 [2.7–15.7)***		3.17 [1.5–6.63]*
	**≥37 month**		**Ref**		Ref
No. children ≤ 5 year	**No child**		Ref		Ref
	**≤ 2**		2.9[1.8–3.8]		2.2 [1.5- 3.23]*
	**> 3**		0.8[0.57- 4.5]		0.89[0.47- 4.6]
Resident	**Rural**			Ref	Ref
	**Urban**			4.5[1.6–8.1] *	14.4[5.19; 41.7] ***
Mass media exposure	**Yes**			**1.2[0.89–1.9]**	1.5 [1.58–3.68]*
	**No**			**Ref**	Ref

*P < 0.05

**P < 0.001

***P < 0.0001

## Discussion

At the end of this study, the prevalence of modern contraceptive utilization among sexually active reproductive-aged women in Amhara region was found 42.4% (38.88–46.15). This report is consistent with previous findings in Harari (40%) [31]. Conversely, this finding is higher than previously reported in Metekel zone (18.6%), in Harari (reported 18.4%), Afar regions(9.8%) [32–34], and national level finding in EDHS 2016 (20.42%) [20], 38.7% pooled report of SSA countries [30], 21% in Ghana(21%), Burkina Faso(24%), Senegal (26.3%), and Sierra Leone(24.9%) [35–39]. The differences could be due to participants’ cultural, religious, and awareness dissimilarity to modern contraceptive methods. Additionally, variations in the study period might contribute to the observed discrepancy in modern contraceptive methods utilization.

Conversely, the final report of this study was lower than earlier reported 58% in Kenya [40], 45·7% in London [35], and 55% in India [36]. The difference could be in the Amhara region, after a girl married and enforced to have a baby soon after marriage before sufficient economy built due to social pressure and more inclined to late to start modern contraceptive utilization [7, 11, 19].

Consistent with previous research findings [8, 30, 41, 42], being age ≤ 25 years, 26–35 years, and 36–45 years have enhanced the odds of modern contraceptive utilization as compared with aged ≥ 46 years. The possible reason might be related to the relationship between the age and educational status of sexually active women; when women were less educated compared to young women and the likelihood of desire for bearing more children increased and less to use family planning [43]. In this report, the odds of utilizing modern contraceptives were 11.6 (AOR = 11.6, 95%CI: 3.22–40.45) for time-enhanced reproductive-aged women who had completed preparatory classes as compared with women who had no formal education. This is consistent with previous findings [1, 16, 30]. When the level of education increases, the likely hood of the desire for bearing more children is decreased and the utilization of family planning increased [4]. This might be linked with youths and adolescents who are eager for sexual activity, but fearful for the outcome of early pregnancy and highly intended to use family planning [40] and intended to be well with their business activities to make their future lives better by extending their childbearing age through contraceptives [43].

The final report of this study revealed that there is a strong relationship between the household economic wealth index and modern contraceptive utilization. Consistent with the previous finding in Ethiopia [4, 8, 30, 32], Nigeria [3], and India [44]; in our report, the odds of modern contraceptive utilization among women in the middle-income class have a significant association with the utilization of modern contraceptives. This might be due to the capacity to purchase modern contraceptives, not necessarily relying on their partners. The wealth variables are an aggregated index of assets, with many having assets such as cell phones, radio, television, and cars, all of which can contribute to important factors to get accessed updated information through different mass media platforms may be challenging for those who are the poorest wealth index class, women.

As evidenced by the multivariable regression of this report, there is a strong relationship between marital status and modern contraceptive utilization. The odds of modern contraceptive utilization were significantly associated with married women as compared with divorced women. This is concordant with earlier reported in Ethiopia [4], and East African countries [8]. The possible reason may be explained as marriage broadens the social bonding and capital base of women, which improves the potential access to media resources through social networks and joint decision-making with the husband and enhances the use of modern contraceptives.

The level of education for sexually active women was an independent predictor for modern contraceptive utilization. In this report, the odds of utilizing modern contraceptives was 11.6 (AOR = 11.6, 95%CI: 3.22–40.45) time enhanced or improved for reproductive-aged women who had completed preparatory classes as compared with no formal education. This is comparable with the previous study finding in Jimma [32], Arbaminch [30], pooled findings in Sub-Saharan Africa [8], Tanzania [42], Mali and Burkina Faso [37]. This might be due to an increase in the level of educational status and increased awareness of sexual and reproductive health, maximizing the probability of getting contraceptive-related information and improving the decision-making ability of women. Meaning, educated women had power in sexual and reproductive health decisions and were well-known about the benefits of modern contraceptives through reading newspapers, mass media, and different social media, and this might help to develop good health care-seeking behaviors including family planning services.

In the final multivariable regression, the odds of women who had ≤ 2 family members in their house were 2.2(AOR = 95%CI: 1.5–3.23) time enhanced to utilized modern contraceptives as compared with no children. This is concordant and similar to the previously reported finding in Addis Ababa [5] and Nigeria [3]. The age of children is one of the prominent factors for the initiation of contraceptive utilization after birth and this is because women who are linked with most lactating women commonly practice the use of contraceptives to reduce the risk of immediate pregnancy and to shape the household structure including the education status, and the limit of several children in a household.

This study indicated that the odds of modern contraceptive utilization methods was 14.4 (AOR = 5.19; 95%CI: 5.19: 41.7) time increased for women who were from urban residents as compared with their counter groups. Unlike previous finding reported in Ethiopia [30], Mali, and Burkina Faso [37]; resident has no significant relation with the utilization of modern contraceptives, however, our report has revealed the odds of utilizing modern contraceptives for sexually active women who reside in an urban area has nearly two-fold increased as compared with women who reside in the rural site. This is consistent with previous findings from the secondary data analysis of the 2016 EDHS [4, 20].

The final report of this study indicated that having mass media exposure to family planning messages had a significant association with contraceptive utilization. This is consistent with pooled reports in Sub Sahara African countries [45], and the Philippines [46]. This might be related to the mass media message about family planning having a significant effect on endorsing and validating negative attitudes about the lesser side effects, issues, and myths of contraceptive use.

All criteria that have been identified as being beneficial, particularly those at the individual level that affect how contemporary contraceptives are used, such as age, education level, and socioeconomic status, reduce maternal fertility rates and hasten the transition of women becoming more empowered and independent. All of these serve as a road map for unified economic growth theory ideas [24, 25].

### Strengths and limitations

The finding of this study has the following strength and limitations during the final reported first of all using advanced models of multilevel regression model removed clustered-level variations and the data set used for the final analysis has highly representative of participants since it was nation level and weighted data. On another other hand, it was impossible to demonstrate a temporal association between the use of contemporary contraceptives and its predictors because of the cross-sectional study design that was employed for the survey. Additionally, because the EMDHS data was a small report, it omitted information on some determinants of the use of contemporary contraceptives.

### Implications for practice

Information dissemination to create awareness about the reassurance of side effects of modern contraceptives for new modern contraceptive users and defaulters is highly ramification to increase or enhance contraceptive utilizers.

## Conclusion

Two out of five reproductive-aged women utilized modern contraceptives in the Amhara region. The use of modern contraceptives is influenced by 21.4% by both socio-demographic and community-level factors of reproductive women. Maternal age, level of education, child age, marital status, and wealth index were identified as individual-level factors which are responsible for the determinants of utilization at individual levels. Information dissemination and reassurance on the side effects of the contraceptive is highly recommended by healthcare providers.

## Data Availability

Data related to this manuscript is available in the hand of the corresponding author and will be obtained under reasonable request.

## Abbreviations

AIC: Akaike’s information criterion
AOR: Adjusted odds ratio
EAs: Enumeration areas
EDHS: Ethiopian Demographic and Health Survey
ICC: Intra-cluster correlation
MOR: Median odds ratio
PCV: Proportional change in variance
